# Neuronal Expression of UBQLN2^P497H^ Exacerbates TDP-43 Pathology in TDP-43^G348C^ Mice through Interaction with Ubiquitin

**DOI:** 10.1007/s12035-018-1411-3

**Published:** 2018-10-30

**Authors:** Vincent Picher-Martel, Laurence Renaud, Christine Bareil, Jean-Pierre Julien

**Affiliations:** 1 Department of Psychiatry and Neuroscience, Laval University, Quebec, Canada; 2 CERVO Brain Research Centre, 2601 Chemin de la Canardière, Québec, QC G1J 2G3 Canada

**Keywords:** Amyotrophic lateral sclerosis (ALS), Ubiquilin-2 (UBQLN2), TAR DNA-binding protein 43 (TDP-43), Animal models, Mice, Neuroinflammation, Ubiquitin

## Abstract

Mutations in the gene encoding ubiquilin-2 (UBQLN2) are linked to amyotrophic lateral sclerosis (ALS) and frontotemporal dementia (FTD). UBQLN2 plays a central role in ubiquitin proteasome system (UPS) and UBQLN2 up-regulation exacerbates TDP-43 cytoplasmic aggregates. To analyze interaction between UBQLN2 and TDP-43 and to produce a relevant ALS animal model, we have generated a new transgenic mouse expressing UBQLN2^P497H^ under the neurofilament heavy (NFH) gene promoter. The UBQLN2^P497H^ mice were then bred with our previously described TDP-43^G348C^ mice to generate double-transgenic UBQLN2^P497H^; TDP-43^G348C^ mice. With low-expression levels of UBQLN2, the double-transgenic mice developed TDP-43 cytosolic accumulations in motor neurons starting at 5 months of age. These double-transgenic mice exhibited motor neuron loss, muscle atrophy, as well as motor and cognitive deficits during aging. The microglia from double-transgenic mice were hyperresponsive to intraperitoneal injection of lipopolysaccharide (LPS). In vivo and in vitro analyses suggested that extra UBQLN2 proteins can exacerbate cytoplasmic TDP-43 accumulations by competing with the UPS for binding to ubiquitin. Thus, increasing the pool of ubiquitin promoted the UPS function with ensuing reduction of TDP-43 cytosolic accumulations. In conclusion, the double-transgenic UBQLN2^P497H^; TDP-43^G348C^ mice provides a unique mouse model of ALS/FTD with enhanced TDP-43 pathology that can be exploited for drug testing.

**Electronic supplementary material:**

The online version of this article (10.1007/s12035-018-1411-3) contains supplementary material, which is available to authorized users.

## Background

Amyotrophic lateral sclerosis (ALS), the most common adult-onset motor neuron disease, is characterized by a progressive loss of the upper and lower motor neurons. ALS evolves with paralysis and the disease is generally fatal within 2 to 5 years after the onset of symptoms. Familial cases, accounting for around 10% of the cases, are caused by mutations in numerous genes. Expanded hexanucleotide repeats in C9orf72 account for almost 40% of the familial cases and other mutated genes include superoxide dismutase 1 (*SOD1*), TAR DNA-binding protein (*TARDBP*)-encoding TDP-43, fused in sarcoma (*FUS*) and ubiquilin-2 (*UBQLN2*) [1]. These mutations can trigger in various pathological changes including axonal transport impairment, excitotoxicity, abnormal RNA processing, neuroinflammation, and protein aggregation [2]. Indeed, pathological diagnosis is generally made with the postmortem observation of motor neurons TDP-43 mislocalization into cytosolic inclusions [3].

TDP-43 is a RNA- and DNA-binding protein mainly localized in the nucleus. It contains nuclear localization and export signals to permit a cytosolic-nuclear shuttling, a glycine rich C-terminal domain and two RNA recognition motifs (RRM1/2). TDP-43 is implicated in RNA transcription, splicing, transport, and stability but also in stress granules formation and in translation [4]. The nuclear loss of TDP-43 and its cytosolic accumulation may participate in ALS pathogenesis by a loss of its normal function [5] or by a gain of new toxic functions [4]. Whether TDP-43 aggregates are toxic and what mechanisms cause TDP-43 aggregation are questions which are not fully resolved. Several proteins are part of the cytosolic TDP-43 inclusions and a defect in their normal cellular role could be part of the explanation. A major component of these aggregates is ubiquilin-2, an ubiquitin-like protein implicated in the ubiquitin proteasome system (UPS) [6]. UBQLN2 is located in TDP-43 aggregates in both familial and sporadic ALS cases and UPS dysfunction is a well-described part of ALS pathogenesis [7]. Mutations in UBQLN2 were also described in other neurological disorders such as frontotemporal dementia (FTD) [6] and spastic paraplegia [8]. Aggregates of UBQLN2 carriers are also associated with p62, FUS, and OPTN, demonstrating its importance in ALS pathology [9, 10].

UBQLN2 connects the UPS and the ubiquitinated proteins. It possesses a PXX domain essential in protein interactions, an N-Terminal ubiquitin-like domain (UBL) which is binding to the 19S complex of proteasome and a C-terminal ubiquitin-associated domain (UBA) binding to Lys^48^-linked chains proteins [11]. We and others have previously shown that overexpression of wild-type and mutant UBQLN2^P497H^ in neuronal cells drives an important TDP-43 mislocalization into cytosolic aggregates [12, 13]. However, the mechanisms for that mislocalization remain unclear. Many reports suggested a proteasome impairment resulting from the expression of mutant UBQLN2 [6, 13–15]. However, it has been suggested that overexpression of both the wild-type and mutant UBQLN2 and other UBL-UBA family members impairs the protein turnover by the proteasome and could result in TDP-43 accumulation by sequestration of proteasome components [11, 16]. Lys^48^-linked ubiquitin chains protein can be directed to the proteasome in an autonomous signal or by the assistance of the UBL-UBA protein family, including UBQLN2. A direct interaction between TDP-43 and UBQLN2 has been demonstrated, but their in vivo significance has not been studied [17].

Animal models are useful to study in vivo pathological mechanisms and to test experimental therapeutic approaches. There are many existing mouse models for ALS research. Mice expressing mutant SOD1 have been widely used but their relevance to sporadic ALS has been questioned. For instance, the mutant SOD1 mice do not exhibit TDP-43 proteinopathy, a hallmark of most ALS cases. Several transgenic mice expressing mutant TDP-43 have been produced but unfortunately, these models do not recapitulate some important features of the disease [1]. They either die before the age of 4–8 weeks, have no motor neuron death, or no TDP-43 proteinopathy [18–21]. The choice of gene promotor and the expression levels of the TDP-43 protein were important factors in mouse phenotypes. Most of these mouse models expressed TDP-43 at higher levels than those detected in human postmortem CNS tissues from ALS cases [22, 23]. In our lab, we have generated different lines of transgenic mice with moderate expression of genomic fragments encoding hTDP-43 with G348C or A315T mutations [24]. These hTDP-43 mice exhibit age-related pathological and behavioral deficits with cytoplasmic hTDP-43 accumulations but without substantial neuronal loss.

Here, we have generated a new mouse model for ALS research based on the co-expression of UBQLN2^P497H^ transgene with mutant TDP-43 transgene. The objective was to exacerbate TDP-43 pathology and ALS features by the expression of two mutant protein with high interactions in the disease and to clarify mechanisms for UBQLN2 pathology. Models with mutation in multiple genes (triple transgenic) represent excellent animal models in other diseases such as Alzheimer’s disease although these mutations are not found together in human [25]. First, we generated a mouse line with neuronal expression of UBQLN2^P497H^ mutant. Second, these UBQLN2^P497H^ mice were crossed with TDP-43^G348C^ mice to generate double-transgenic UBQLN2^P497H^; TDP-43^G348C^ mice. Interestingly, these double-transgenic mice developed typical features of ALS/FTD including TDP-43 proteinopathy, motor neuron loss, muscle atrophy, gliosis, and motor and cognitive impairment.

## Methods

### Generation of Transgenic Mice

A 2.9 kb fragment of the human neurofilament heavy chain hNFH promoter was subcloned into the pBluescript KS+ plasmid (pBSKS-hNFH) using Sal1 restriction site in C-terminal and KPN1 in N-terminal. Oligonucleotides are described in Table 1. The Flag-UBQLN2^P497H^ fragment was obtained by PCR from our previously described UBQLN2 plasmid [12] and was introduced into the pBSKS-hNFH plasmid. The plasmid sequence was verified by repeated sequencing. Using BssHI restriction site for linearization, a 5.2 kb fragment containing hNFH-flagUBQLN2^P497H^ construct was isolated on agarose gel and micro-injected into mice one-cell embryos having a background of C57Bl/6. Transgenic mice were genotyped with flag amplification by PCR from tail samples DNA. Only one mouse line was identified from the founders and was bred with non-transgenic C57Bl/6 to establish a stable transgenic line. The line was named UBQLN2^P497H^ mice. The transgenic TDP-43^G348C^ mice were generated as previously described [24]. It should be noted that TDP-43^G348C^ gene was driven by its endogenous promoter. The two mice lines were crossed and the resulting double-transgenic mice were called UBQLN2^P497H^; TDP-43^G348C^. Four groups were utilized for analysis: non-transgenic (NTG), UBQLN2^P497H^, TDP-43^G348C^, and UBQLN2^P497H^; TDP-43^G348C^ double-transgenic mice. Experiments were performed between 5 and 18 months of age. All experiments were performed on age-matched littermates. Groups of mice were sacrificed at 5, 8, 12, and 18 months of age, and each group was separated into equal number of males and females.

**Table 1:** Oligoprimers used for plasmid construction and q-RT-PCR

Gene	Forward primer (5′–3′)	Reverse primer (5′–3′)
Flag human UBQLN2	GGGACGACGAATTCGAGGCAGC ATGGACTACAAGGACGACGATGA	GGGACGACGCGGCCGCTTACGAT GGCTGGGAGCCCAG
Human NFH promoter	GGGACGACGGTACCCTAGGACAT TCTGGGCTGAGATC	GGGACGACGTCGACCAGCGGAG CGGGAGTGCGGGGCT
Human UBQLN2 (Q-RT-PCR)	TGCCGCGGGAACTAACACTAC	GGAGCTCAGAGAAGTTGGTCGA
Mouse UBQLN2 (Q-RT-PCR)	CTCCACACCTACCACCACGAATA	GCTGCTGCATCTGGTTCTGAAG
GAPDH (Q-RT-PCR)	GGCTGCCCAGAACATCATCCCT	ATGCCTGCTTCACCACCTTCTTG

### Quantitative Real-Time PCR

Tissues were homogenized in Qiazol buffer (Qiagen, Germantown, MD, USA), and total RNA was extracted using RNeasy mini kit on column DNase (Qiagen, Hilden, DE). First-strand cDNA was synthesized using superscript IV Rnase H-RT (Invitrogen Life Technologies) and purified with Qiagen purification kit. Oligonucleotides designed with GeneTool (Table 1) were used to perform fluorescent-based real-time PCR quantification. Number of copies for each mRNA were calculated using second derivate method and a standard curve base on known amounts of purified PCR products. Number of copies were normalized on levels of GAPDH mRNA.

### Accelerating Rotarod

Accelerating rotarod was performed on mice at 3 rpm speed with 0.1 rpm/s acceleration. Twenty mice per group were analyzed. Mice were trained three times in the first week and then tested every week from 5 months of age to 18 months of age. The mean score from three trials was used for statistical analysis. To facilitate the comparison between each mouse, we used the relative rotarod score. Because we observed that mice were reaching their best capacity around 6 months of age (suppl file 3 g), we used the score at 6 months of age as the basal level for each mouse. Every week, the score was compared to this basal score for each mouse and we showed the results in percentage of best score.

### Step-Through Passive Avoidance Test

Passive avoidance test is based on the natural tendency of the mice to prefer dark chamber. The device (Ugo Basile company) is composed of a bright compartment separated from a dark compartment by a guillotine door. The mice were tested at 5 and 7 months of age with a 3-day protocol. The first day, the mouse is placed into the bright room and allowed to explore the room for 30 s before the door opens. The second day, the mouse is also place in the bright room but received an electric shock when it transferred to the black room (0.6 mA, 2 s). On the third day (48 h later), the door opens after 5 s and we recorded the time it takes to enter the dark room. Mice with memory deficits are rapidly going to the dark room. At 7 months of age, only the first day was measured to verify if mice remember receiving the shock 2 months earlier.

### Pole Test

We constructed a 50-cm pole with a gripping rope. Mice were putted on the top of the pole, and latency time to descend the pole was measured. We performed two trials by mouse at 15 months of age. We also analyzed the control of the mice when descending the pole. For that purpose, we divided the pole into four sections. We started at 100% of control and removed 25% for every section without control.

### Cat-Walk Analysis

Home-made cat-walk corridor was constructed using a 60 cm by 7 cm one-sided open ramp. Paper was deposed in the corridor. Mice foots were painted with non-toxic paint and the mice were then deposed in the corridor. Mice had a natural tendency to walk toward the open-side of the corridor. We used a different color for forelimb and hindlimb paw. We measured both stride length and base of support of the paw. Mice were tested at 12 and 18 months of age.

### Mice Sacrificed, Protein Extraction, and Immunoblotting

Mice were either sacrificed at 5, 8, or 12 months of age for molecular and pathological analysis. Mice were first intraperitoneally injected with pentobarbital 12 mg/ml (0.1 ml/10 g of body weight) for anesthesia. Blood was removed from the mice by slow intracardiac perfusion of saline 1 ml/g of body weight. The brain and spinal cord were removed and snap-frozen in liquid nitrogen for protein extraction. In case of immunofluorescence, mice were perfused with saline followed by 4% paraformaldehyde and kept in 4% paraformaldehyde for 24 h hours before long-time conservation in sucrose.

Cytoplasmic and nuclear extraction was performed on mice brains and spinal cords. Tissues were homogenized in hypotonic buffer (10 mM HEPES-KOH pH 7.6, 10 mM NaCL, 1 mM KH_2_PO_4_, 5 mM NaHCO_3_, 5 mM EDTA pH 8.0, 1 mM CaCl_2_, 0.5 mM MgCl_2_, and 1X protease and phosphatase inhibitor cocktail). The lysis was incubated on ice for 10 min. 2.5 M sucrose was added and tissues were homogenized again. After a 10-min centrifugation at 6300*g*, supernatant was kept as the cytoplasmic fraction. The pellet was resuspended in TSE buffer (10 mM Tris pH 7.4, 300 mM sucrose, 1 mM EDTA, 0.1% NP-40, and 1X protease and phosphatase inhibitor cocktail). Resuspended pellet was centrifuged at 4000*g* for 5 min and this step was repeated three times for washing. Finally, pellet was resuspended in RIPA buffer (50 mM Tris pH 7.4, 1 mM EDTA, 150 mM NaCl, 2% SDS, 1% NP-40, and 1X protease and phosphatase inhibitor cocktail) and then sonicated.

Total protein fraction was obtained using RIPA buffer (50 mM Tris pH 7.4, 1 mM EDTA, 150 mM NaCl, 0.25% SDS, 1% NP-40, 0.1 mM DTT, and 1X protease and phosphatase inhibitor cocktail). Tissue lysis was then sonicated and centrifuged at 14000 rpm for 10 min. Insoluble and soluble extracts were prepared according to previously described methods [12]. Forty micrograms of proteins were loaded into 10% SDS-page gels for all immunoblotting analysis. Antibodies used were TARDBP (Proteintech, Chicago, 1:2500), UBQLN2 (Abcam, Cambridge, 1:1000), actin (Milipore, Etobicoke, 1:20000), p84 nuclear matrix (Abcam, Cambridge, 1:1000), GAPDH (Santa-cruz, Dallas, 1:1000), hTDP-43 (Abnova, Taiwan, 1:1000), phospho-TDP-43 (Sigma Aldrich, Oakville, 1:1000), flag M5 (Sigma Aldrich, Oakville, 1:1000), Iba1 (Wako, Richmond, 1:1000), GAL3 (Abcam, Cambridge, 1:1000), GFAP (Cell signaling, Whitby, 1:1000), phospho-NF-κB p65 (Cell signaling, Whitby, 1:1000), NF-κB p65(Santa-cruz, Dallas, 1:1000), and lys48-ubiquitin (Millipore, Etobicoke, 1:1000).

### Immunofluorescence and Immunohistochemistry

Fixed tissues were cut using microtome into 25-μm sections and kept in antifreeze. For immunofluorescence, epitope retrieval was performed using boiling citrate buffer for 10 min. Sections were then blocked with 10% goat serum for 1 hour, and first antibody was incubated overnight at 4 °C. Secondary antibody was incubated 1 hour at room temperature. Primary antibodies were TARDBP (Proteintech, Chicago, 1:500), SQSTM1/P62 (Cell signaling, Whitby, 1:200), UBQLN2 (Abcam, Cambridge, 1:200), CHAT (Millipore, Etobicoke, 1:200), GFAP (Cell signaling, Whitby, 1:500), Iba1 (Wako, 1:500), ubiquitin (Millipore, Etobicoke, 1:500), phosphoTDP-43 (Sigma Aldrich, Oakville, 1:200), and Tia1 (Santa Cruz, Dallas, 1:500). Secondary antibodies were alexa-fluor goat anti-rabbit (1:500), alexa-fluor goat anti-mouse (1:500), and alexa-fluor anti-goat (1:500). For immunohistochemistry, sections were fixed with xylene and rehydrated with decreasing concentration of ethanol (95, 70, 50). Sections were washed with PBS and endogenous peroxidase attenuation was performed with 0.6% H_2_O_2_. Tissues were again washed with PBS 0.25% triton X-100 and then blocked with 10% goat serum-PBS-triton for 1 h at room temperature. Primary antibodies (TDP-43, proteintech, 1:500 and UBQLN2, Abcam, 1:200) were incubated overnight at 4 °C and then slices were washed with PBS-triton. Biotinylated secondary antibodies (Vector company, Burlingame) were incubated for 2 h at room temperature and slices washed again with PBS-triton. ABC complex (Vectastain, Burlingame, USA) was incubated for 60 min. DAB staining was applied for 5 min, and counterstain with hematoxylin was performed for 1 min. Finally, sections were dehydrated with increasing concentration of ethanol (70, 90, 95, 100, 100) and fixed with xylene.

### H&E Staining and Ventral Root Preparation

Gastrocnemius muscle was collected when mice were sacrificed (see Mice Sacrificed, Protein Extraction, and Immunoblotting section). Muscles were transversally cut with cryostat. Muscle were then post-fixed during 5 min with 4% PFA. Muscle slice were washed with decreasing concentration of ethanol (90%, 70%, and 50%) during 1 min for every wash. Tissues were rehydrated with MQ water for 1 min. Hematoxylin (Sigma Aldrich, Oakville) was added for 6 min and then washed with tap water until tissue was clear of superfluous hematoxylin. Tissue were treated with 70% acid ethanol to increased hematoxylin fixation and then cleaned for 1 min with MQ water. Eosin (Sigma Aldrich, Oakville) was added to the tissue for 30 s and also cleaned with tap water until the excess eosin was removed. Tissue was then dehydrated with increasing concentration of ethanol (50%, 70%, 90%, and 100%) for 1 min each. The sliced were finally treated with xylene.

Dorsal root ganglia (DRGs) (containing both ventral and dorsal root) were collected when mice were sacrificed. DRGs were post-fixed with 4% PFA for 1 day and keep in 3% glutaraldehyde solution until preparation. DRGs were washed three times during 5 min with NaHPO_4_ 0.1 M pH 7.4 and incubated in osmium tetroxide 2% (Canemco-Marivac 0163) in NaHPO_4_ 0.2 M during 2 h at room temperature. After NaHPO_4_ 0.1 M pH 7.4 washes, tissues were dehydrated with ethanol (50%, 70%, 90%, 100%, 50/50 ethanol/acetone and acetone) and then with 50/50 acetone/epoxy resin during 1 h at room temperature. Tissues were embedded with epoxy resin (Marivac TAAB 812, DDSA, NMA, DMP30 from Canemco-Marivac) overnight at room temperature and cooked at 60 °C for 24 h.

### Cell Culture and Transfection

All cell culture experiments were performed with the mouse neuroblastoma cell line Neuro2A. Cells were cultured in Dulbecco’s Modified Eagle Medium (DMEM) with 10% fetal bovine serum (FBS), 1% penicillin-streptomycin, and 1% glutamine. We used lipofectamine 2000 as a transfection reagent. Twenty-four hours after transfection, opti-MEM was replaced by growth media and kept for another 24 h. We used HA-ubiquitin plasmid (Addgene #18712, Cambridge, USA) containing single-coding sequence of the ubiquitin B (UBB) gene in the pcDNA3 backbone. We also used previously described pCMV-hUBQLN2^P497H^ [12] and pCMV-hTDP-43^G348C^ [23] plasmids. We took advantage of pCDNA3 plasmid as control. Cells were collected at 48 h after transfection. Cytoplasmic and nuclear fractionation was performed according to previous described methods [12]. For immunofluorescence experiments, cells were fixed with 4% PFA and methanol and blocked with 10% goat serum before primary antibody (see immunofluorescence section).

### Immunoprecipitation

Proteins were extracted from the mice brain as previously described in the Mice Sacrificed, Protein Extraction, and Immunoblotting section. Dynabeads protein G (Invitrogen, Norway) were washed twice with 1x PBS-tween 0.02% and resuspended in PBS-tween. Ubiquilin-2 (Abcam) antibody was added to the beads and incubated at room temperature for 1.5 h. Antibody-binded beads were washed two times with PBS-tween and with buffer containing 0.1 mg/ml BSA, 10 mM Hepes, and 1x PBS. Three hundred micrograms of proteins were incubated overnight at 4 °C with the antibody-binded beads. Beads were washed twice with BSA-Hepes-PBS buffer and washed once with hepes-PBS buffer. Proteins were first eluted with SSB and boiling at 95 °C for 7 min and again with DTT at 95 °C for 5 min. Proteins were directly loaded to the immunoblotting gel.

### Lipopolysaccharide Treatment and Cytokines Array

Mice were intraperitoneally injected with 5 mg/kg lipopolysaccharide (LPS) at 8 months of age. Tissue were collected 24 h later and processed as described in protein extraction. Cytokine array was performed according to mouse cytokine antibody array C4 protocol (RayBiotech, Norcross, USA). Two mice per group were combined in the same sample for the array. Gel were scanned and intensity from each cytokine spots was measured using ImageJ software. Results were normalized on positive control spots intensity in each membrane.

### Proteasome Assay

We used luciferase chymotrypsin-like assay kit from Promega Company. This luminescent assay measured chymotrypsin-like protease activity associated with the UPS. The assay is based on the presence of the UPS substrate succinyl-leucine-leucine-valine-tyrosine-aminoluciferin (Suc-LLVY-aminoluciferin). Following cleavage by the proteasome, the aminoluciferin is released from Suc-LLVY-aminoluciferin and is transformed into luminescent signal by the included recombinant luciferase. The luminescent signal is proportional to the UPS function in the samples. We adapted our protocol using previously described protocol from muscle extract and suggested protocol from the company [26]. Both cells and mouse tissues were managed with the same protocol. Cells/tissues were snap-frozen in liquid nitrogen. We destroyed cellular membrane using PBS-0.05% NP-40 followed by sonication. Samples were centrifuged at 4000*g* for 5 min. We quantified protein content in supernatant and dilute the samples to obtain a concentration of 0.4 μg/μl. We then used 50 μl (20 μg) of each sample and mixed it with 50 μl of the proteasome-glo reagent in a 96-well white plate. Samples were analyzed in triplicate and one out of three triplicates was treated with proteasome inhibitor MG-132. Luciferase activity was measured using Enspire reading machine at 60 min. Luciferase activity with MG-132 inhibitor was subtracted for each duplicate to remove non-specific signals.

### Statistical Analysis

We used *t* test and one-way ANOVA when appropriate for statistical analysis.

#### Availability of Data Materials

All data generated or analyzed during this study are included in this published article and its supplementary information files.

## Results

### Generation of UBQLN2^P497H^ Mutant Mice

**Fig. 1:**
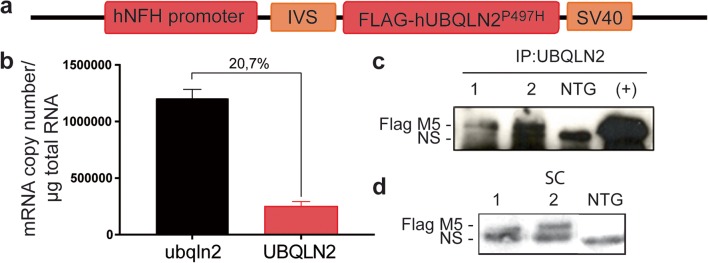
UBQLN2^P497H^ transgenic mouse line generation. **a** Schematic representation of the construct with flag-UBQLN2^P497H^ gene driven by hNFH promoter. IVS, intervening sequence; SV40 polyA. **b** Quantitative reverse-transcriptase PCR of the mouse and human UBQLN2 mRNAs in the brain of UBQLN2^P497H^ mice at 5 months of age (*n* = 3). **c** Immunoprecipitation of UBQLN2 from the brain extracts of two transgenic UBQLN2^P497H^ mice, one non-transgenic mouse (NTG), and Neuro2A cells transfected with pCMV-hUBQLN2^P497H^ plasmid (positive control) (NS non-specific band). **d** Flag immunoblotting of spinal cord (SC) from two UBQLN2^P497H^ mice [1, 2] and one non-transgenic mouse (NTG) at 5 months of age (NS non-specific band)

cDNA-encoding full-length flag-tagged P497H mutant human ubiquilin-2 (UBQLN2) gene was cloned into a plasmid containing the neuronal-specific NFH gene promoter (Fig. 1a). The construct was micro-injected in C57Bl/6 one-cell embryos. We identified 13 mice founders bearing the transgene. For unknown reason, only one founder transmitted the transgene to its offspring and we used this mouse founder for line generation. The levels of transgene mRNA expression were measured using qRT-PCR on three mice from the F1 generation. The mRNA level from the UBQLN2 transgene corresponded to 20.7% the level of endogenous mouse Ubqln2 (Ubqln2) (Fig. 1b). The extra levels of ubiquilin-2 in the UBQLN2 ^P497H^ transgenic mice are of physiological relevance for human cases. The flagged UBQLN2^P497H^ protein was detected by immunoprecipitation of the brain and spinal cord extracts with anti-flag antibody followed by SDS-PAGE fractionation and immunoblotting (Fig. 1c). The flagged protein was also detected by immunoblotting after SDS-PAGE of the brain or spinal cord extracts (Fig. 1d). As expected, the flagged UBQLN2^P497H^ was not detected in other tissues (suppl Fig. 1a). The mice had a normal development and normal survival.

### TDP-43 Proteinopathy in Double-Transgenic Mice

**Fig. 2:**
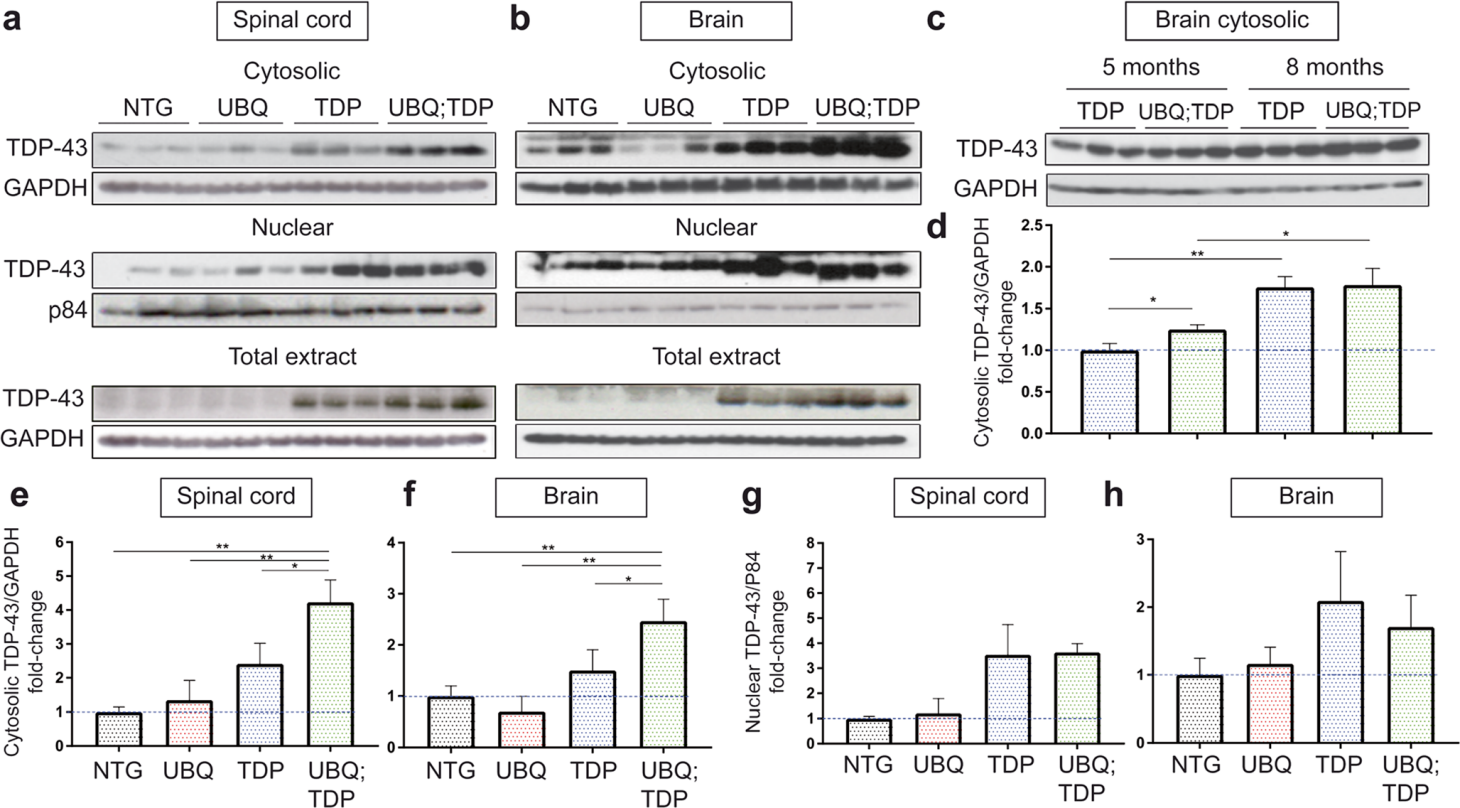
Cytosolic TDP-43 accumulations in double-transgenic mice. **a** Cytosolic, nuclear, and total protein extracts from the spinal cord of non-transgenic (NTG), UBQLN2^P497H^ (UBQ), TDP-43^G348C^ (TDP), and double-transgenic UBQLN2^P497H^; TDP-43^G348C^ (UBQ; TDP) mice (*n* = 3, age = 5 months). **b** Cytosolic, nuclear, and total protein extracts from the brain (*n* = 3, age = 5 months). **c** Cytosolic fraction from the brain of TDP-43^G348C^ and double-transgenic mice at 5 months and 8 months of age (*n* = 3). **d** Quantification of TDP-43 levels vs GAPDH levels in the cytosolic fractions of the brain at 5 and 8 months of age. **e** Quantification of the TDP-43 levels vs GAPDH levels in the cytosolic fraction of spinal cord (*n* = 3, age = 5 months). **f** Quantification of the TDP-43 levels vs GAPDH levels in the cytosolic fraction of brain (*n* = 3, age = 5 months). **g** Quantification of the TDP-43 levels vs P84 nuclear matrix levels in the nuclear fraction of the spinal cord (*n* = 3, age = 5 months). **h** Quantification of the TDP-43 levels vs P84 nuclear matrix levels in the nuclear fraction of the brain (*n* = 3, age = 5 months)

We crossed UBQLN2^P497H^ mice with TDP-43^G348C^ mice [24] to generate double-transgenic UBQLN2^P497H^; TDP-43^G348C^ mice (suppl Fig. 1b). Age-matched non-transgenic littermates were used as a control group. To characterize TDP-43 pathology in double-transgenic mice, we fractionated cytosolic and nuclear fractions from the spinal cord and the brain. We observed a 1.75-fold increase in level of TDP-43 in cytosolic fractions of the spinal cord from double-transgenic mice as compared to single-hTDP-43^G348C^ mice (*p* = 0.0251) and 4.23-fold increased as compared to NTG mice (*p* = 0.0012) at 5 months of age (Fig. 2a, e). No increase of TDP-43 in the cytosolic fraction was detected in UBQLN2^P497H^ single transgenic. An increased in level of TDP-43 was also present in the brain cytosolic extract of double-transgenic mice as compared to TDP-43^G348C^ mice (1.64-fold, *p* = 0.0468) and to NTG mice (2.47-fold, *p* = 0.0057) (Fig. 2b, f). We detected no significant changes in the level of TDP-43 in the nuclear fractions or in the level of TDP-43 in total extract from the brain and spinal cord between TDP-43^G348C^ and double-transgenic mice (Fig. 2a–h). Furthermore, we detected an increase in levels of the truncated 35 kDa form of TDP-43 in the brain cytosolic extract of double-transgenic mice as compared to NTG (3.5-fold, *p* = 0.0418) and a non-significant increase as compared to TDP-43^G348C^ mice (1.8-fold, *p* = 0.2729) (suppl Fig. 2a, b). In conclusion, the cytosolic fraction of TDP-43 was significantly increased in double-transgenic mice, as compared to other groups in both the brain and spinal cord at 5 months of age.

We noted that the cytosolic TDP-43 levels increased between 5 and 8 months of age in TDP-43^G348C^ (1.754-fold, *p* = 0.001) and in double-transgenic (1.424-fold, *p* = 0.0118) (Fig. 2c, d). Both TDP-43^G348C^ mice and double-transgenic mice exhibited similar TDP-43 levels in the brain cytosolic fractions TDP-43 at 8 months of age. These results suggest that low expression of UBQLN2 can precipitate mislocalization of TDP-43 and TDP-43 also start to mislocalize around 8 months of age in TDP-43^G348C^ mice, as previously described [24]. The same amount of cytosolic TDP-43 at 8 months of age could also be explained by the death of motor neurons with abundant cytosolic TDP-43 levels in double transgenic (Fig. 4a, b).

**Fig. 3:**
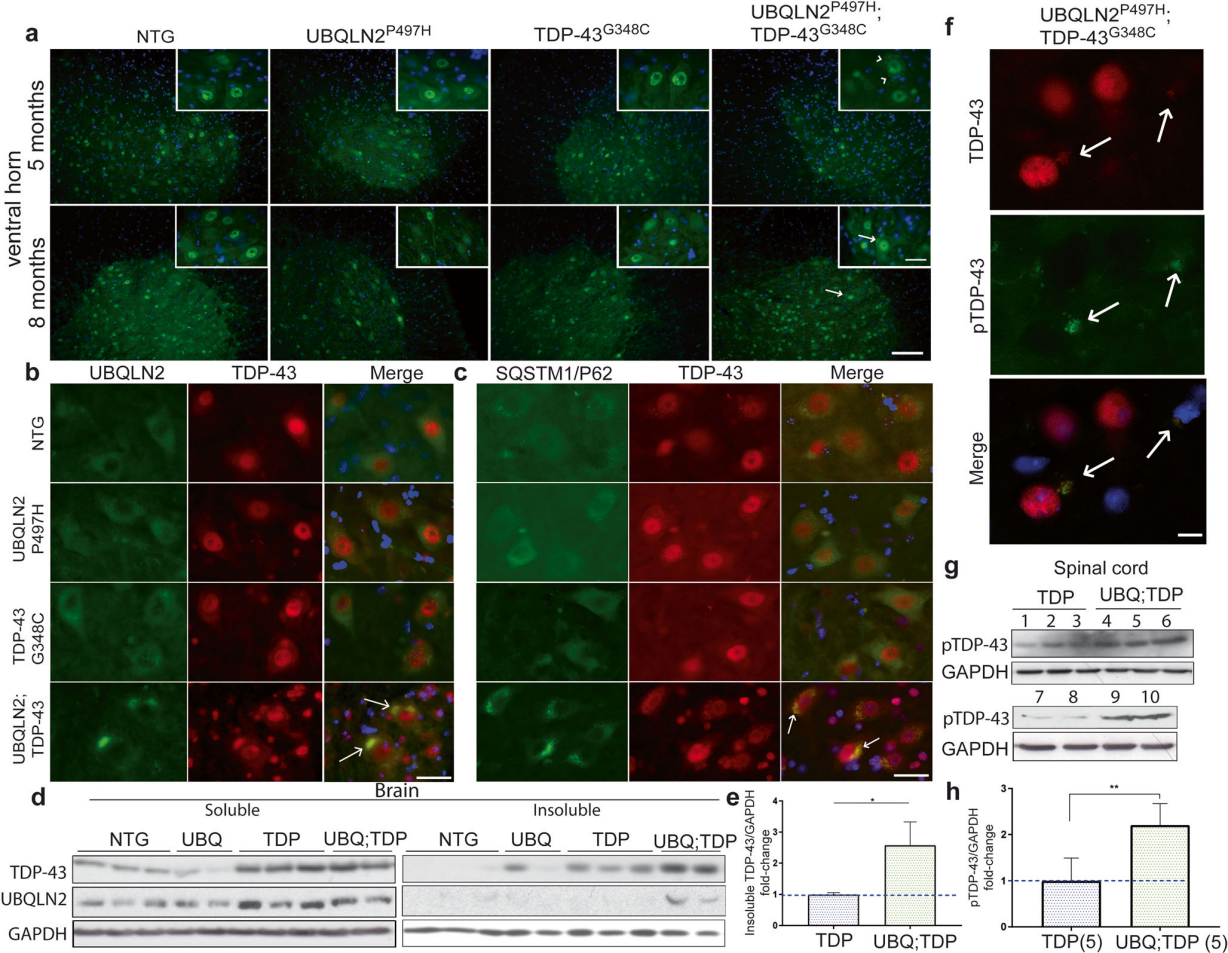
TDP-43/UBQLN2 inclusions at 8 months of age. **a** Immunofluorescence of lumbar ventral horn at 5 and 8 months of age using TDP-43 antibody at × 20 and × 40 magnification (scale bar = 100 μm and 25 μm respectively) (*n* = 3). **b** Co-immunofluorescence of lumber ventral horn at × 40 magnification using UBQLN2 and TDP-43 antibodies (scale bar = 25 μm) (*n* = 3). **c** Co-immunofluorescence of lumbar ventral horn at × 40 magnification using SQSTM1/P62 and TDP-43 antibodies (scale bar = 25 μm) (*n* = 3). **d** Brain soluble-insoluble protein extracts from 8 months old non-transgenic (NTG, *n* = 3), UBQLN2^P497H^ (UBQ, *n* = 2), TDP-43^G348C^ (TDP, *n* = 3), and double-transgenic UBQLN2^P497H^; TDP-43^G348C^ (UBQ; TDP, *n* = 2) mice. **e** Quantification of the TDP-43 levels vs GAPDH levels in the brain insoluble extracts. **f** Co-immunofluorescence of lumbar ventral horn at × 100 magnification using TDP-43 and phospho-TDP-43 antibodies (scale bar = 10 μm). **g** Total protein extracts from spinal cord of five 8 months old TDP-43^G348C^ [1–3, 7, 8] and five double-transgenic UBQLN2^P497H^; hTDP-43^G348C^ mice [4–6, 9, 10]. **h** Quantification of the pTDP-43 levels vs GAPDH levels in the spinal cord. White arrow = TDP-43 inclusions (8 months), arrow head = UBQLN2 punctate signals (5 months)

To further validate the effects of UBQLN2^P497H^ on TDP-43 cytosolic accumulation, we performed immunofluorescence detection of TDP-43 on tissue sections from the spinal cord ventral horn, hippocampus, and cortex at 5 months and 8 months of age. Interestingly, the double-UBQLN2^P497H^; TDP-43^G348C^ mice at 5 months of age exhibited punctate TDP-43 signals in cytoplasm of spinal motor neurons (Fig. 3a). Such punctate signals of TDP-43 were not detected in motor neurons of NTG mice and in single-transgenic, UBQLN2^P497H^, or TDP-43^G348C^ mice. Since both TDP-43 and UBQLN2 are implicated in stress granules formation and degradation [27, 28], we performed co-immunofluorescence to detect if these punctate signals could be stress granules. Using TIA1, a well-described stress granules marker [28, 29], we observed co-labeling of TDP-43 and TIA1 in the spinal cord of double-transgenic mice (suppl Fig. 2c). This could suggest that UBQLN2 and TDP-43 are colocalized in stress granules in early pathologic state of double-transgenic mice.

Neither cortex nor hippocampus exhibited cytosolic TDP-43 inclusions at 5 months of age **(**suppl Fig. 1c–d**)**. Cytosolic TDP-43 inclusions became evident in the ventral horn at 8 months of age in double-transgenic mice (Fig. 3b, c). The TDP-43 inclusions in double-transgenic mice were positive for Ubqln2 and Sqstm1/P62 (Fig. 3b, c). It is noteworthy that more TDP-43/DAPI co-staining signals surrounded motor neurons in double-transgenic mice as compared to other single-transgenic mice or NTG mice (Fig. 3b, c). This is explained by gliosis surrounding motor neurons (see section on Gliosis and Inflammatory Profiles, Fig. 6**)**. TDP-43 inclusions were also found in the cortical and hippocampal neurons of double-transgenic mice at 8 months of age. The presence of TDP-43 and UBQLN2 inclusions in the cytoplasm of neurons in the brain and spinal cord of double-transgenic mice was also confirmed by immunohistochemistry (suppl Fig. 1e-i).

To quantify TDP-43 aggregation, we performed soluble/insoluble fractionation with high-speed centrifugation and sonication previously described in [12]. The samples were then subjected to SDS-PAGE and immunoblotting with antibody against TDP-43. The results revealed a significant increase of insoluble TDP-43 (2.58-fold, *p* = 0.0287) in the brain of double-transgenic mice at 8 months of age (Fig. 3d, e) as compared to single-TDP-43^G348C^ mice which started to exhibit TDP-43 aggregates at 10 months of age [24]. Insoluble UBQLN2 was detected only in double-transgenic mice (Fig. 3d). Inclusions of phosphorylated TDP-43 are a hallmark of ALS in postmortem CNS samples [30]. As shown in Fig. 3f, inclusions containing phosphorylated-TDP-43 were also detected by immunofluorescence microscopy with antibodies against TDP-43 and phospho-TDP-43. A twofold increase (*p* = 0.004, *n* = 5) in levels of phospho-TDP-43 were also detected in the spinal cord of double-transgenic mice as compared to hTDP-43^G348C^ mice (Fig. 3g, h). Taken together, these results suggest that the expression of hUBQLN2^P497H^ has triggered the formation of cytosolic inclusions containing insoluble TDP-43 species.

### Motor Neuron Loss, Muscle Atrophy, and Axonal Degeneration in Double-Transgenic Mice

**Fig. 4:**
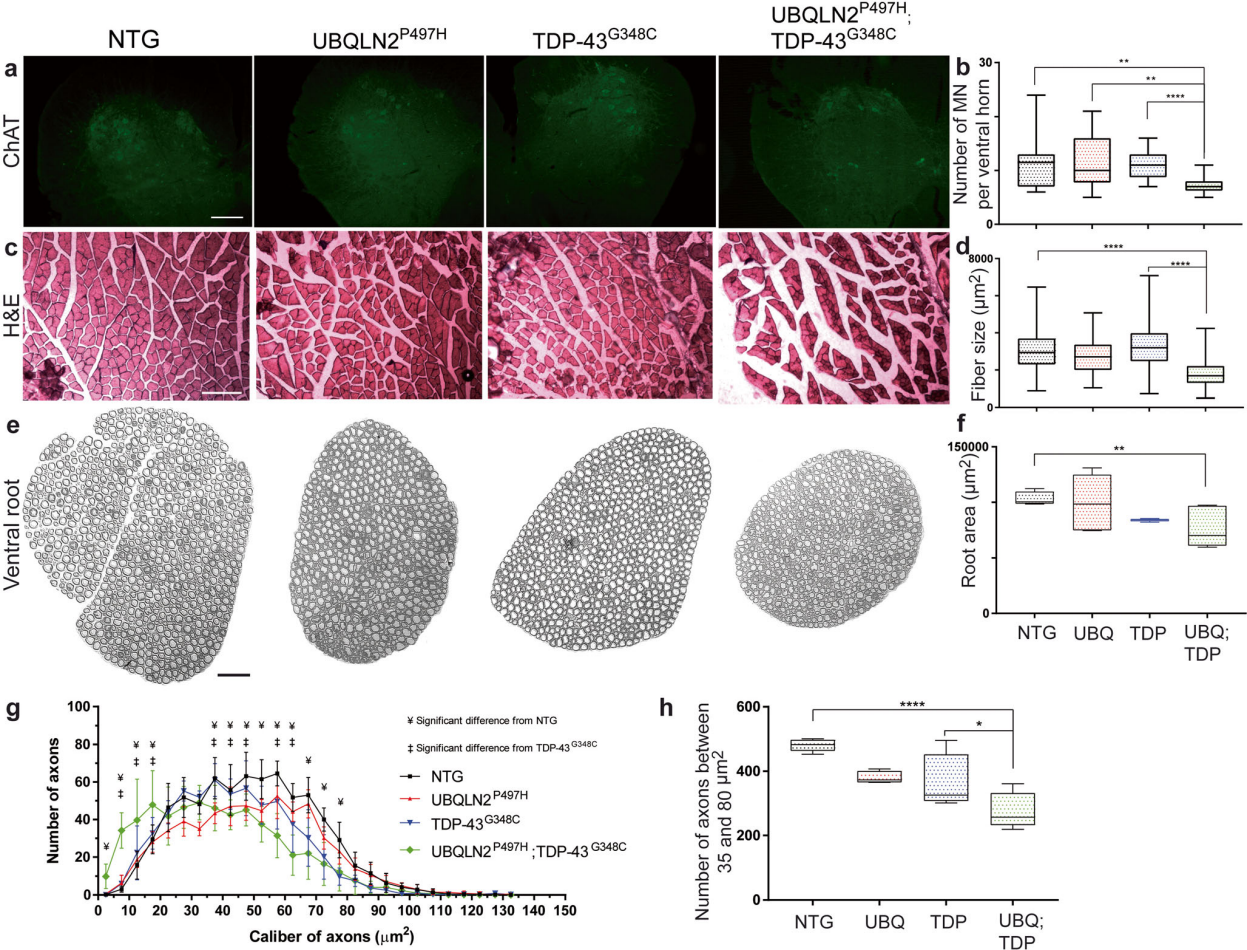
Motor neuron loss, axonal degeneration, and muscle atrophy. **a** Immunofluorescence of ChAT in lumbar ventral horn at 8 months of age. ChAT staining is specific to motor neuron. Pictures were taken at × 20 magnification (scale bar = 100 μm). **b** Number of motor neuron (MN) per ventral horn (three mice, six sections per mice). **c** Hematoxylin & eosin (H&E) coloration of transverse section of gastrocnemius muscle at 8 months of age. Pictures were taken at × 10 magnification with light microscopy (scale bar = 200 μm). **d** Muscle fiber size in μm^2^ (three mice, three sections per mice. **e** Epoxy-fixed ventral root innervating gastrocnemius muscle at 8 months of age. Pictures were taken at × 20 magnification with light microscopy (scale bar = 100 μm). **f** Total ventral root area in μm^2^ (three mice, three sections per mice). Area were all manually measured with ImageJ software. **g** Number of axons per caliber interval in μm^2^. Only significant results between double transgenic and NTG (¥, *p* ˂ 0.05) and between double transgenic and TDP-43^G348C^ are shown (‡, *p* ˂ 0.05). Results are also significant between double transgenic and UBQLN2^P497H^ (not illustrated). **h** Number of large axons between 35 and 80 μm^2^ combined in one interval

To quantify motor neurons in the spinal cord, we performed immunostaining of choline acetyltransferase (ChAT) in ventral horn of mice at 8 months of age. We used three animals per group and six slices of the lumbar spinal cord per animal. Results revealed a 33% loss of the motor neurons in double-transgenic mice as compared to TDP-43^G348C^ mice (*p*˂0.0001) (Fig. 4a, b). Neither the TDP-43^G348C^ mice nor the UBQLN2^P497H^ exhibited significant loss of the motor neurons during aging [24]. Microscopy of transverse sections of gastrocnemius muscle stained with hematoxylin and eosin (H&E) also revealed severe muscle atrophy in the double-transgenic mice at 8 months of age (Fig. 4c). A reduction of 48% in the muscle fiber size was measured in double-transgenic mice as compared to TDP-43^G348C^ mice (*p*˂0.0001) (Fig. 4d). The motor neuron loss was also confirmed by the analysis of axonal size in the L5 ventral root from the transgenic mice. There was a significant 26% (*p* = 0.0002) shrinking in the size of ventral roots of the double-transgenic mice as compared to NTG mice (Fig. 4e, f). The number of large caliber axons (35 μm^2^ to 80 μm^2^) was reduced by 25% (*p* = 0.0195) in double transgenic as compared to TDP-43^G348C^ and by 43% (*p* ˂ 0.0001) as compared to normal NTG mice **(**Fig. 4g, h**)**. Note that there was a significant increase in small caliber axons which may reflect in part the shrinking of large axons during neurodegeneration process or compensatory regrowth of small motor axons. More studies would be needed to clarify this phenomenon.

### Motor and Cognitive Deficits in Double-Transgenic Mice

**Fig. 5:**
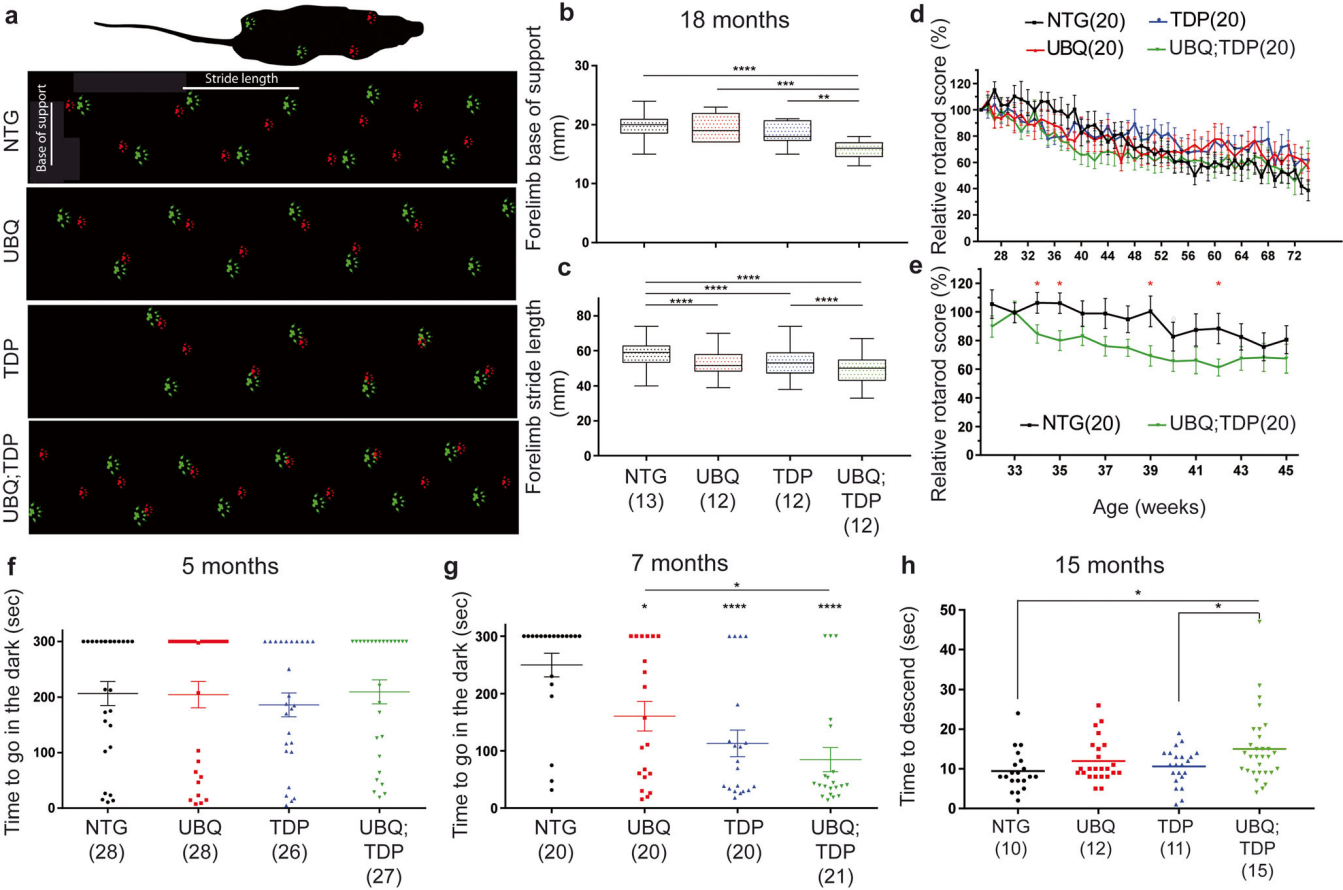
Motor and cognitive impairment in double-transgenic mice. **a** Representative figure of the cat-walk analysis at 12 months of age. Hindlimb paw is in red and forelimb paw in green. Stride length is the distance between paw from the same limb and base of support is the distance between the two hindlimbs or the two forelimbs. **b** Forelimb base of support at 18 months of age. Number of mice per group in parentheses. **c** Forelimb stride length at 18 months of age. Results are similar in hindlimb analysis (see suppl Fig. 3). **d** Rotarod score from 5 months of age to 18 months of age. Results are shown in relative score as compared to best mice capacity. **e** Rotarod score from 33 weeks of age to 45 weeks of age for non-transgenic (NTG) and double transgenic (UBQ; TDP). These are the only time point with significant differences. **f** Passive avoidance test represented in time to go in the dark at the third day (testing day) at 5 months of age and at 7 months of age in (**g**). **h** Pole test analysis at 15 months of age. Time to descend the pole was measured in seconds

Various tests were used for analyses of motor phenotypes. First, the cat-walk analysis was performed using a 60-cm corridor. We measured the stride length of the paws and the base of support of the mice (Fig. 5a). There was no significant difference in the forelimb base of support at 12 months of age in double-transgenic mice (14.78 ± 0.7027 mm) as compared to TDP-43^G348C^ (15 ± 0.7868 mm, *p* = 0.8365) (suppl Fig. 3a), but the reduction was significant at 18 months of age (16 ± 0.4606 mm and 18.58 ± 0.543 respectively, *p* = 0.0015) (Fig. 5b). We observed a reduced forelimb stride length in double-transgenic mice from 12 months of age (57.19 ± 0.7995 mm) as compared to TDP-43^G348C^ (63.25 ± 1.045 mm, *p* ˂ 0.0001) (suppl Fig. 3d), but this reduction was more prominent at 18 months of age (49.43 ± 0.6357 mm and 53.56 ± 0.6797 mm respectively, *p* ˂ 0.0001) (Fig. 5c). The results were similar in hindlimb analysis except that results were significant in base of support from 12 months of age (UBQ: TDP = 25.22 ± 0.7778 and TDP-43^G348C^ = 28.71 ± 1.426, *p* = 0.0386) (suppl Fig. 3b). These results may reflect that double-transgenic mice have difficulty to extend their legs.

Second, we performed the pole test analysis at 15 months of age. Mice were deposed on a 50-cm pole and the descent time was measured. We measured an increased in the descent time of double-transgenic (15.02 ± 1.662 s) mice as compared to TDP-43^G348C^ mice (10.59 ± 0.9884, *p* = 0.0382) and NTG (9.45 ± 1.134, *p* = 0.0146) (Fig. 5h).

Third, we did not observe major differences between all four groups in the rotarod test (Fig. 5d). This lack of significant results could be explained by the lower weight of the double-transgenic mice (suppl Fig. 3i). Indeed, the high-weighted NTG and TDP-43^G348C^ fell faster in the rotarod test which could have affected results. However, we denoted significant differences in the rotarod score from 32 weeks to 45 weeks of age between NTG mice and double-transgenic mice (Fig. 5e). At this age, the weight difference was less important. Finally, double-transgenic mice also exhibited a reduced gastrocnemius muscle weight at 12 months of age (suppl Fig. 3h).

To assess the cognitive performance of the mice, we performed the passive avoidance test. This test is based on natural tendency of the mice to prefer the dark room and is used to analyze memory impairment. Using a 3-day protocol (habituation, shock, and testing), we observed no memory impairment in the double-transgenic mice at 5 months of age (Fig. 5f). However, at 7 months of age, the double transgenic exhibited marked memory impairment (84.71 ± 21.15 s, *p* ˂ 0.0001) when compared to TDP-43^G348C^ mice (113.1 ± 23.37 s, *p* ˂ 0.0001), UBQLN2^P497H^ mice (160.7 ± 25.95 s, *p* = 0.0104) and to NTG mice (249.8 ± 20.46 s) (Fig. 5g).

### Gliosis and Inflammatory Profiles

**Fig. 6:**
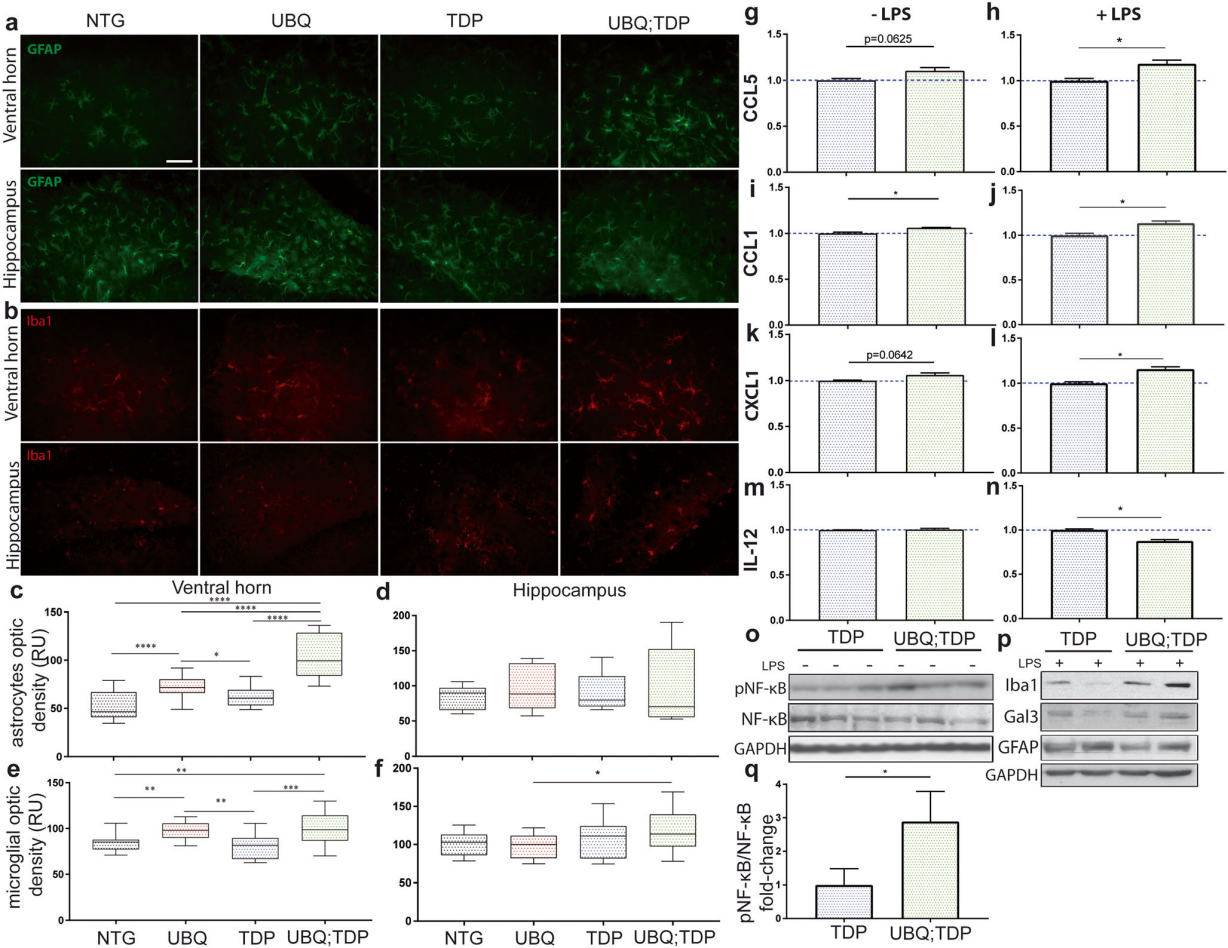
Gliosis and susceptibility to NF-κB-driven inflammation. **a** GFAP immunofluorescence of the ventral horn and hippocampus at 8 months of age (scale bar = 50 um, magnification = × 40). **b** Iba1 immunofluorescence of the ventral horn and hippocampus at 8 months of age. **c** GFAP signal measured with optic density in six different fields per mice in the ventral horn and **d** hippocampus (*n* = 3 mice per group). **e** Iba1 signal measured with optic density in six different fields per mice in the ventral horn and **f** hippocampus (*n* = 3 mice per group) (RU = random unit). Cytokines array levels of **g–h** CCL5, **i–j** CCL1, **k–l** CXCL1, **m–n** IL-12 in brain extracts of transgenic mice (TDP-43^G348C^ and UBQLN2^P497H^; TDP-43^G348C^ in blue and green, respectively) 24 h after intraperitoneal injection of LPS (*n* = 2) of without injection of LPS (*n* = 3). Only cytokines with significant changes are illustrated here. **o** Immunoblotting and SDS-PAGE analysis of the total brain extracts from non-injected mice (*n* = 3) and **p** the total brain extracts from LPS injected mice (*n* = 2). **q** Quantification of the pNF-κB levels vs NF-κB levels from brain extracts (*n* = 3)

We performed immunofluorescence to measure the gliosis. At 5 months of age, no microgliosis or astrogliosis was detected in the hippocampus, cortex, and lumbar spinal cord in any mouse groups (suppl Fig. 4). However, at 8 months of age, we observed an increased signal of microglial staining with Iba-1 antibody in the ventral horn of the double-transgenic mice as compared to TDP-43^G348C^ (1.25-fold, *p* = 0.0009) and NTG (1.19-fold, *p* = 0.002) (Fig. 6b, e). Microglia staining with Iba-1 antibody was also slightly increased in the single-UBQLN2^P497H^ mice as compared to TDP-43^G348C^ (1.21-fold, *p* = 0.0012) and NTG mice (1.16-fold, *p* = 0.0012). Nonetheless, microgliosis was more prominent in the double-transgenic mice than in the single-UBQLN2^P497H^ transgenic mice (Fig. 6e). In the hippocampus, only a significant increase was measured in double transgenic as compared to UBQLN2^P497H^ single transgenic (1.21-fold, *p* = 0.0439) (Fig. 6f). Astrocytes staining was also increased in the spinal cord ventral horn of double-transgenic mice at 8 months of age as compared to TDP-43^G348C^ mice (1.65-fold, *p* ˂ 0.0001), UBQLN2^P497H^ mice (1.41-fold, *p* ˂ 0.0001) and NTG mice (1.92-fold, *p* ˂ 0.0001) (Fig. 6a, c). Again, an increased in astrocytes signal in UBQLN2^P497H^ mice was noted as compared to TDP-43^G348C^ (1.17-fold, *p* = 0.0156) and NTG mice (1.37-fold, *p* ˂ 0.0001) (Fig. 6c). No difference between the mouse group was observed in GFAP immunostaining in the hippocampus (Fig. 6d).

To further characterize the inflammatory profile of single- and double-transgenic mice, we performed cytokines arrays and immunoblotting analyses with or without intraperitoneal injection of lipopolysaccharide (LPS) at 8 months of age. LPS is a known microglial cells activator and is widely used to study microglial profiles in vivo [31, 32]. By SDS-PAGE and immunoblotting, we observed an increased in the Iba1 and Gal3 levels in the brain of double-transgenic mice as compared to TDP-43^G348C^ mice when injected with LPS, meaning that microglia are more activatable (Fig. 6p). There were no significant changes without LPS injection (data not shown). However, there was no difference in GFAP signal between groups after LPS injection (Fig. 6p). Using cytokines array, we analyzed the inflammatory profiles in the brain of LPS-treated mice. Briefly, proteins were extracted from the brain samples and incubated with membranes containing antibody against many cytokines. After extensive washing, the signal for each cytokine is measure with developing films. We observed no significant change for most cytokines profiles. However, we observed a small significant increase in levels of CCL5 (*p* = 0.0324), CCL1 (*p* = 0.0280), and CXCL1 (*p* = 0.0200) in double-transgenic mice as compared to TDP-43^G348C^ (Fig. 6h–l**)** as well as a reduction in IL-12 levels (*p* = 0.0127) (Fig. 6n). We also compared the cytokine profiles of double-transgenic and TDP-43^G348C^ mice without LPS injection. As in LPS-treated mice, most cytokines levels were similar in the single- and double-transgenic mice. However, we observed a significant increase in CCL1 levels (*p* = 0.0272) (Fig. 6i) in double-transgenic mice and an almost significant increase in CCL5 levels (*p* = 0.0625) (Fig. 6g) and CXL1 (*p* = 0.0642) (Fig. 6k). There was no difference in IL-12 levels (*p* = 0.6852) (Fig. 6m).

CCL5 is a strong chemo-attractant secreted by activated lymphocytes and monocytes, a result of NF-κB pathway activation [33]. It is increased in serum and CSF of sALS patients [34]. CCL1 is secreted by the monocytes or macrophages in response to LPS exposure to promote the NF-κB pathway, and CXCL1 is also implicated in NF-κB activation through the PI3K/AKT signaling pathway and is also increased in ALS patient’s fibroblasts [35, 36]. We have previously reported that UBQLN2 up-regulation can activate the NF-κB signaling pathway [12]. Here, by SDS-PAGE and immunoblotting of brain samples, we observed a significant increase in phospho-NF-κB levels in double-transgenic mice as compared to TDP-43^G348C^ mice (2889 ± 0.5194-fold, *p* = 0.0327) (Fig. 6o, q).

### hUBQLN2^P497H^ Increased TDP-43 Cytosolic Accumulation through Ubiquitin Sequestering

To investigate the mechanism underlying the induced TDP-43 mislocalization in double-transgenic mice, we hypothesized that expression of UBQLN2^P497H^ may sequester ubiquitin proteins with ensuing reduction of TDP-43 degradation by the UPS.

**Fig. 7:**
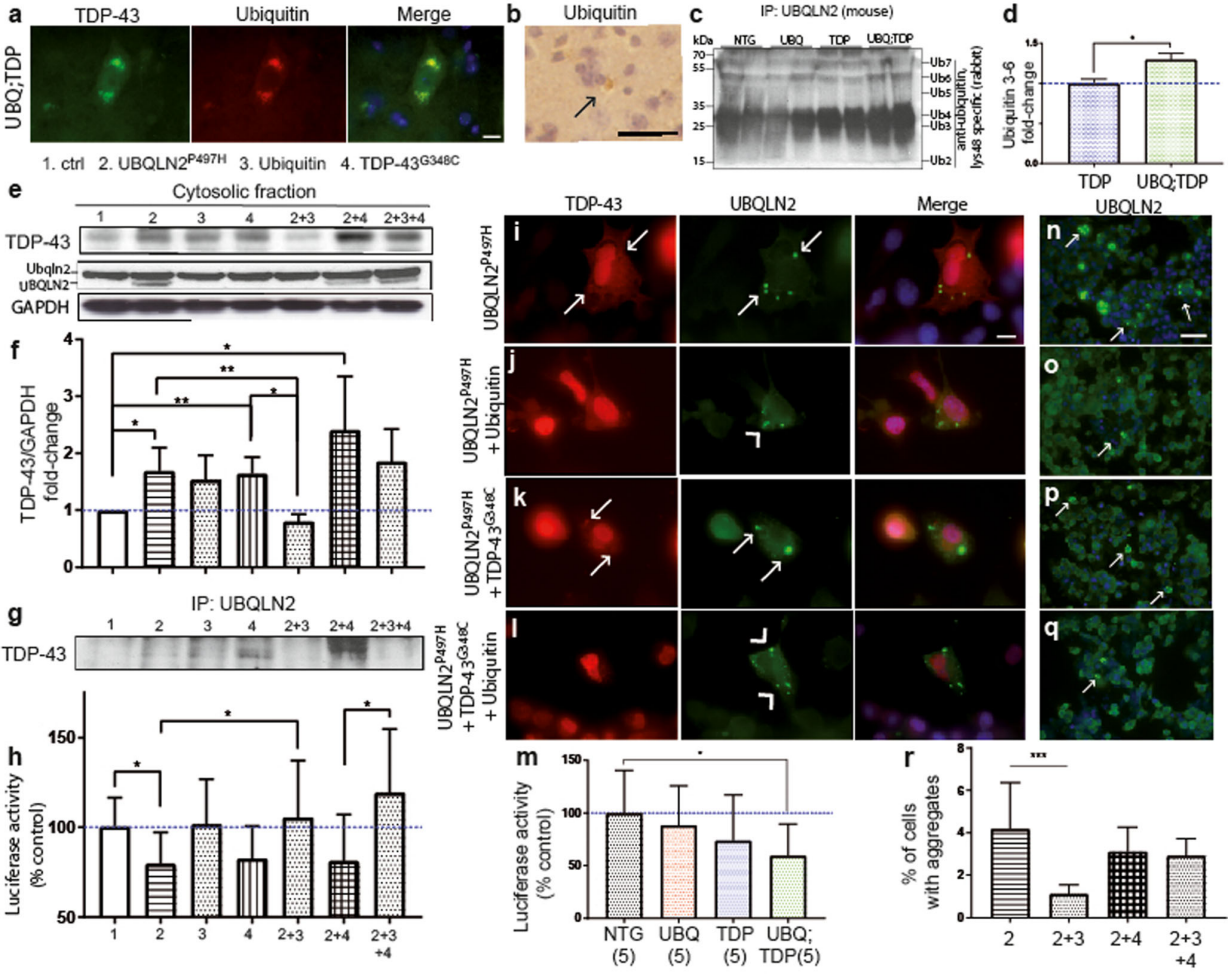
Reversal of UBQLN2-driven UPS dysfunction and TDP-43 aggregation by upregulation of ubiquitin**. a** TDP-43 and ubiquitin co-immunofluorescence in spinal cord of double-transgenic mice at × 100 magnification (scale bar =10 μm). **b** Ubiquitin immunohistochemistry from cortex of double-transgenic mice at 8 months of age at × 40 magnification (scale bar = 100 μm, black arrow = ubiquitin inclusion). **c** Immunoprecipitation of UBQLN2 from brain of transgenic mice at 8 months of age followed by SDS-PAGE and immunoblotting with Lys^48^-bound specific antibody (Ub ubiquitin 2–7) to detect co-immunoprecipitated ubiquitin chain. **d** Quantification of the ubiquitin 3, 4, 5, and 6 levels after immunoprecipitation of UBQLN2 from brain extracts of TDP-43^G348C^ and UBQLN2^P497H^; TDP-43^G348C^ mice. **e** TDP-43 and UBQLN2 detection by immunoblotting of cytoplasmic extracts from Neuro2A transfected cells 48 h after transfection with either control (1), pCMV-hUBQLN2^P497H^ (2), pCMV-hTDP-43^G348C^ (4), pCMV-ubiquitin (3), or a combination of these plasmids. **f** Quantification of the TDP-43 levels in the cytosolic fraction of the Neuro2A-transfected cells (*n* = 3). **g** Co-immunoprecipitation of TDP-43 with UBQLN2 extracts from Neuro2A-transfected cells. **h** Proteasome-glo chymotrypsin-like assay in the Neuro2A-transfected cells with either control, pCMV-hUBQLN2^P497H^, pCMV-hTDP-43^G348C^, pCMV-ubiquitin of a combination of these plasmids (*n* = 5). **i–l** TDP-43 and UBQLN2 co-immunofluorescence in Neuro2A-transfected cells at × 100 magnification (arrow head = TDP-43 negative inclusions, arrow = TDP-43 positive inclusions) (scale bar = 10 μm). **m** Proteasome-glo chymotrypsin-like assay with spinal cord extracts from transgenic mice (*n* = 5). **n–q** UBQLN2 immunofluorescence of Neuro2A-transfected cells 48 h after transfection with same combination of plasmids (white arrow = UBQLN2 aggregates). Pictures were taken at × 40 magnification (scale bar = 50 μm). **r** Percentage of cells with aggregates. Pictures were taken on six fields per group and total number of cells were count with Dapi staining

First, we confirmed that ubiquilin-2 interacted with ubiquitin in the double-transgenic mice. With immunofluorescence and immunohistochemistry, we observed that ubiquitin proteins were colocalized with cytoplasmic TDP-43 in double-transgenic mice at 8 months of age in the spinal cord (Fig. 7a, b). We also performed an immunoprecipitation of UBQLN2 in the brain extracts from the four groups of mice. This was followed by SDS-PAGE and immunoblotting with Lys^48^-linked ubiquitin antibody. As shown in Fig. 7c, higher levels of Lys^48^-linked ubiquitin proteins co-immunoprecipitated with UBQLN2 in samples from the double-transgenic mice as compared to TDP-43^G348C^ (1.3-fold, *p* = 0.0478) (Fig.7d). This suggested an increased sequestering of Lys^48^-linked ubiquitin chains by UBQLN2 in double-transgenic mice.

Strategies to increase ubiquitination may favorize the UPS function and help to protect against TDP-43 toxicity [37, 38]. To further determine the effects of ubiquitin up-regulation on TDP-43 cytosolic accumulation, pCMV expression vectors coding for UBQLN2^P497H^, ubiquitin, and TDP-43^G348C^ were transfected alone or in combination into Neuro2A cells. As shown in Fig. 7e, f, expression of pCMV-UBQLN2^P497H^ (1.7-fold, *p* = 0.0148, *n* = 3) or pCMV-TDP-43^G348C^ (1.6-fold, *p* = 0.0045, *n* = 3) led to an increase in levels of TDP-43 in the cytosolic fraction. An additional increase in cytoplasmic TDP-43 levels was detected in cells co-transfected with both pCMV-UBQLN2^P497H^ and pCMV-TDP-43^G348C^ (2.4-fold, *p* = 0.0241, *n* = 3) (Fig. 7e, f). Conversely, there was a reduction in levels of cytosolic TDP-43 in cells co-transfected with pCMV-ubiquitin in combination with either pCMV-UBQLN2^P497H^ (53%, *p* = 0.0061) or pCMV-TDP-43^G348C^ (51%, *p* = 0.0149). In case of triple transfection with pCMV-UBQLN2^P497H^, pCMV-TDP-43^G348C^, and pCMV-ubiquitin, the reduction in TDP-43 was not significant (23%, *p* = 0.35) (Fig. 7e, f). Immunoprecipitation of UBQLN2 in transfected cells revealed increased binding of TDP-43 with UBQLN2 when UBQLN2 [2] or TDP-43 was up-regulated [4] and when both proteins were up-regulated (2 + 4) (Fig. 7g). Indeed, when ubiquitin was also up-regulated, we observed a reduced binding of UBQLN2 with TDP-43 in cells (2 + 3 and 2 + 3 + 4) (Fig. 7g). The UBQLN2 loading used for immunoprecipitation was equivalent (Fig. 7e). This could mean that TDP-43 is degraded or is removed from aggregates by reduced binding with UBQLN2.

The level of cytoplasmic UBQLN2 was decreased in cells co-transfected with pCMV-UBQLN2^P497H^ and pCMV-ubiquitin as compared to pCMV-UBQLN2^P497H^ alone (2 and 2 + 3) (Fig. 7e). Consistently with results in Fig. 7r, this could mean that cytosolic accumulation of UBQLN2 into aggregates was reduced with ubiquitin up-regulation. However, when TDP-43 is also up-regulated (2 + 3 + 4), UBQLN2 is still accumulated into the cytosol, but without the presence of TDP-43 (Fig. 7e).

Immunostaining of UBQLN2 in transfected cells (Fig. 7i–q) revealed large number of ubiquilin-2 aggregates in Neuro2A cells expressing pCMV-UBQLN2^P497H^ and in cells co-expressing pCMV-UBQLN2^P497H^ and pCMV-TDP-43^G348C^ (Fig. 7i–p) However, a reduction in percentage of cells with ubiquilin-2 aggregates occurred in cells co-transfected with pCMV-UBQLN2^P497H^ and pCMV-ubiquitin plasmids (1.1%, Fig. 7n, r) as compared to pCMV-UBQLN2^P497H^ alone (4.2%) (*p* = 0.0003) **(**Fig. 7o, r). We also observed a reduction in TDP-43 cytoplasmic aggregates co-localizing with ubiquilin-2 inclusions in cells co-transfected with pCMV-ubiquitin and pCMV-UBQLN2^P497H^ (Fig. 7j) as compared to pCMV-UBQLN2^P497H^ transfected cells **(**Fig. 7i). Similarly, expression of pCMV-ubiquitin led to a decrease of TDP-43 cytoplasmic aggregates in cells co-expressing pCMV-UBQLN2^P497H^ and pCMV-TDP-43^G348C^ (Fig. 7k. l). These results suggest that increasing the pool of ubiquitin proteins may compensate for ubiquitin sequestering by ubiquilin-2. This would in turn promote the UPS function with ensuing reduction in levels of TDP-43 cytosolic accumulations.

It is well established that proteasome inhibition may cause TDP-43 cytoplasmic accumulation [39–42]. Because we observed reduced TDP-43 cytosolic accumulation with ubiquitin up-regulation, we used a chymotrypsin-like assay to measure the proteasome efficacy in these transfected cells. We observed a significant reduction in proteasome activity in cells transfected with pCMV-UBQLN2^P497H^ (80%, *p* = 0.0191, *n* = 5), a significant reduction in cells transfected with pCMV-TDP-43^G348C^ (83%, *p* = 0.0424, *n* = 5), and a reduction in cells transfected with both plasmids (81%, *p* = 0.0764, *n* = 5) as compared to control cells (Fig. 7h). The presence of ubiquitin up-regulation corrected the proteasome dysfunction in cells transfected with pCMV-UBQLN2^P497H^ (*p* = 0.0481, *n* = 5) and in cells co-transfected with pCMV-UBQLN2^P497H^; pCMV-TDP-43^G348C^ (*p* = 0.0189, *n* = 5). We also used the same protocol on the spinal cord extract from transgenic mice and observed a significant reduction of 40% in the proteasome function of double-transgenic mice (*p* = 0.0203, *n* = 5) (Fig. 7m). These results suggested that a proteasome impairment by UBQLN2^P497H^ up-regulation may participate to TDP-43 accumulation in double-transgenic mice.

## Discussion

Here, we report for the first time the generation and characterization of a double-transgenic mice co-expressing two distinct ALS-linked genes UBQLN2^P497H^ and TDP-43^G348C^ mutations. The use of the NFH promoter with low expression led to modest neuronal expression levels of UBQLN2^P497H^. Thus, the UBQLN2^P497H^-transgenic mice exhibited a 20% extra level of ubiquilin-2 in the brain. Whereas, the single-UBQLN2^P497H^ transgenic mice did not developed robust behavioral phenotypes, except slight cognitive deficits and mild TDP-43 accumulation in the hippocampal neurons, the double-transgenic mice developed many behavioral and pathological features reminiscent of ALS/FTD. First, the double UBQLN2^P497H^; TDP-43^G348C^ transgenic mice exhibited an age-related TDP-43 proteinopathy with cytosolic TDP-43 mislocalization and inclusion bodies containing phospho-TDP-43, ubiquitin, and p62 in the spinal cord, cortex, and hippocampus. Second, at 8 months of age, the double-transgenic mice exhibited a 33% loss of the motor neurons which was accompanied by severe muscle atrophy and degeneration of large myelinated axons in the ventral roots. Third, double-transgenic mice developed motor impairment phenotypes at cat-walk analysis and pole test. Significant reduction in rotarod score was observed between 32 and 45 weeks of age in the double transgenic. Fourth, the double-transgenic mice developed cognitive deficits measured by the passive avoidance test at 7 months of age, while lower extent of cognitive deficits occurred in single-transgenic UBQLN2^P497H^ and TDP-43^G348C^ mice. Fifth, the double-transgenic mice exhibited astrogliosis and microgliosis in the ventral horns of the lumbar spinal cord.

Other transgenic mice expressing UBQLN2 mutants have been reported recently. Our model has some particularities when compared to other described UBQLN2 models. The discrepancies between models could be much explained by the levels of transgene expression. One mouse model expressing UBQLN2^P497H^ under the control of hUBQLN2 promoter developed cognitive deficits, ubiquilin-2 inclusions in the hippocampus, and a dendritic spinopathy [14]. In this model, the level of expression of human UBQLN2 was similar to the level of endogenous mouse ubiquilin-2, and this study did not report TDP-43 pathology and loss of motor neurons. Another study reported a knocked-in hUBQLN2^P506T^ mice which exhibited cognitive deficits but without motor deficits [43]. Due to the knock-in approach, the UBQLN2 level was normal. A third animal model, a transgenic UBQLN2^P497H^ rat with TRE-doxycycline system exhibited UBQLN2 aggregates and hippocampal/cortex neuronal death at the age of 130 days [44]. Finally, a recent study reported motor neuron loss, TDP-43 pathology, and cognitive deficits in Thy1.2-driven UBQLN2^P497S^ or UBQLN2^P506T^ transgenic mice [45]. In these mice, the expression levels of transgenes were approximately 70–80% of the endogenous mouse Ubqln2. However, cytoplasmic TDP-43 accumulations were not examined in these models. The phenotypes were variable in the animal cohorts. For instance, only 10% of the UBQLN2^P497S^ mice and 40% of the UBQLN2^P506T^ mice developed hindlimb paralysis.

There are lines of evidence suggesting that pathogenic pathways may arise not only from the mutations in UBQLN2 but also from the overexpression of WT UBQLN2. For instance, transgenic mice overexpressing WT hUBQLN2 exhibited loss of upper motor neurons and hippocampal neurons, albeit the loss was less pronounced than in the UBQLN2 mutant lines [45]. Likewise, rats bearing WT UBQLN2 transgene had similar deficits and pathology than transgenic UBQLN2^P497H^ rats [46]. Furthermore, our cell culture studies revealed that both WT and P497H variant of UBQLN2 can trigger TDP-43 aggregation and cell death [12]. Thus, the combined results suggest that an increase in expression levels of the WT UBQLN2, such as those reported here in Fig. 1, may contribute to pathogenesis in sporadic ALS.

Our double-transgenic mouse model suggested an important synergic effect of UBQLN2 and TDP-43 in ALS pathology [17]. Our results with cultured cells suggested that overexpressed UBQLN2 may sequester Lys^48^-bound ubiquitin with ensuing reduction in proteasome efficacy (Fig. 7). These results are consistent with a report more than a decade ago suggesting that the overexpression of UBQLN2 homolog (named RAD23A) may compete with the proteasome for binding to polyubiquitinated proteins [16]. The UBA domain of UBQLN2, which binds to Lys^48^-bound ubiquitin is necessary for that competition [13, 47]. Several reports also proposed that either mutation in UBQLN2 or overexpression of WT UBQLN2 reduced the UPS function [6, 13–15]. The impact of overexpressed UBQLN2 with several mutations on the UPS function was compared in vitro [15]. The P497H mutation was linked to the weaker proteasome impairment but it caused more protein aggregation than other mutations. Removing of the PXX domain containing the mutation had no effect on toxicity and UBQLN2 protein accumulation [13]. A recent report also suggested that huntingtin and ataxin-3 can sequester UPS machinery through ubiquitin-UBA binding in aggregates [48]. However, mutation in the UBQLN2 PXX domain reduced binding to heat shock protein (HSP) and it reduced clearance of protein aggregates [43]. These studies suggest that mutations in UBQLN2 may promote the formation of aggregates, but they are not a requirement for aggregation. In sporadic ALS, a slight increase in UBQLN2 levels in context of a deficit in clearance pathway may contribute to TDP-43 accumulation [41]. Here, our studies with transfected cultured cells revealed that increase in levels of ubiquitin protein can reduce the TDP-43 cytosolic accumulation and aggregation through enhancement of the UPS function. This mechanism may represent a therapeutic avenue.

Recently, UBQLN2 has been linked to stress granules degradation via the implication of ubiquitin [27]. The overexpression of ubiquitin reduced the UBQLN2 levels in stress granules by driving it to the proteasome [27]. In Fig. 3a, we observed punctate signals resembling stress granules in double-transgenic mice. These signals co-localized with the stress granules marker TIA1 (suppl Fig. 2c). Our results could suggest that the up-regulation of UBQLN2 may recruit mutated TDP-43 and TIA1 in stress granules and favorize their progression into inclusions. Indeed, we previously observed that UBQLN2/TDP-43 inclusions are dynamic structures which combine to become large inclusions [12], similar to the stress granules’ dynamic [49]. However, more work must be completed to clarify this hypothesis.

It is now well established that neuroinflammation can contribute to ALS progression [2] but this has been studied mainly in context of disease caused by SOD1 mutations [32, 50, 51]. The double-transgenic UBQLN2^P497H^; TDP-43^G348C^ mice described here might be useful to study the role of neuroinflammation in context of TDP-43/UBQLN2 pathology. Our results suggest that microglia in these double-transgenic mice are more prone to inflammation and become hyperactivated by lipopolysaccharide (LPS) exposure. The double-transgenic mice exhibited higher levels of phosphorylated NF-κB in the brain as compared to single TDP-43^G348C^ mice even though expression of the UBQLN2^P497H^ transgene was restricted to neurons (Fig. 6o). Actually, both UBQLN2 and TDP-43 can contribute to enhance NF-κB activation [23]. Moreover, when treated with LPS, the double-transgenic mice exhibited higher levels of CCL1, CXCL1, and CCL5 cytokines compared to single TDP-43^G348C^ mice (Fig. 6). It has been recently demonstrated that the extracellular presence of truncated or mutant forms of TDP-43 act on CD14 receptor to induce NF-κB activation in microglial cells [52]. NF-κB activation results in cytokines secretion and microglial-dependant motoneuron death. Our results could suggest that UBQLN2-drived TDP-43 pathology may promote microglial activation through this mechanism. However, this remains to be demonstrated in vivo.

## Conclusions

In conclusion, our results suggest that the co-expression of two ALS-linked proteins in mice, UBQLN2^P497H^ and TDP-43^G348C^, exerted synergistic effects in the development of TDP-43 pathology as well as cognitive and motor deficits. Based on our cell culture studies, we propose that neuronal expression of UBQLN2^P497H^ may alter UPS function via sequestering of ubiquitin, thereby promoting cytoplasmic proteinopathy with formation of TDP-43 aggregates. This unique double-transgenic mouse with ALS/FTD-like features offers an interesting model for testing potential therapeutic approaches to target TDP-43 proteinopathy.

## Electronic supplementary material

ESM 1 (JPG 5603 kb)

ESM 2 (JPG 193 kb)

ESM 3 (JPG 249 kb)

ESM 4 (JPG 966 kb)

## Abbreviations

UBQLN2: human ubiquilin-2
Ublqn2: mouse ubiquilin-2
ALS: amyotrophic lateral sclerosis
FTD: fronto temporal dementia
UPS: ubiquitin proteasome system
TDP-43: TAR DNA-binding protein 43
NFH: neurofilament heavy
LPS: lipopolysaccharide
UBL: ubiquitin-like domain
UBA: ubiquitin-associated domain
NTG: non-transgenic
DRG: dorsal root ganglia
DMEM: Dulbecco’s Modified Eagle Medium
H&E: hematoxylin and eosin
