# Supplementary figures and images for: Neuronal Expression of UBQLN2P497H Exacerbates TDP-43 Pathology in TDP-43G348C Mice through Interaction with Ubiquitin
Source: Mol Neurobiol. 2018 Oct 30;56(7):4680–96. doi: 10.1007/s12035-018-1411-3 (PMC6647471; doi:10.1007/s12035-018-1411-3)

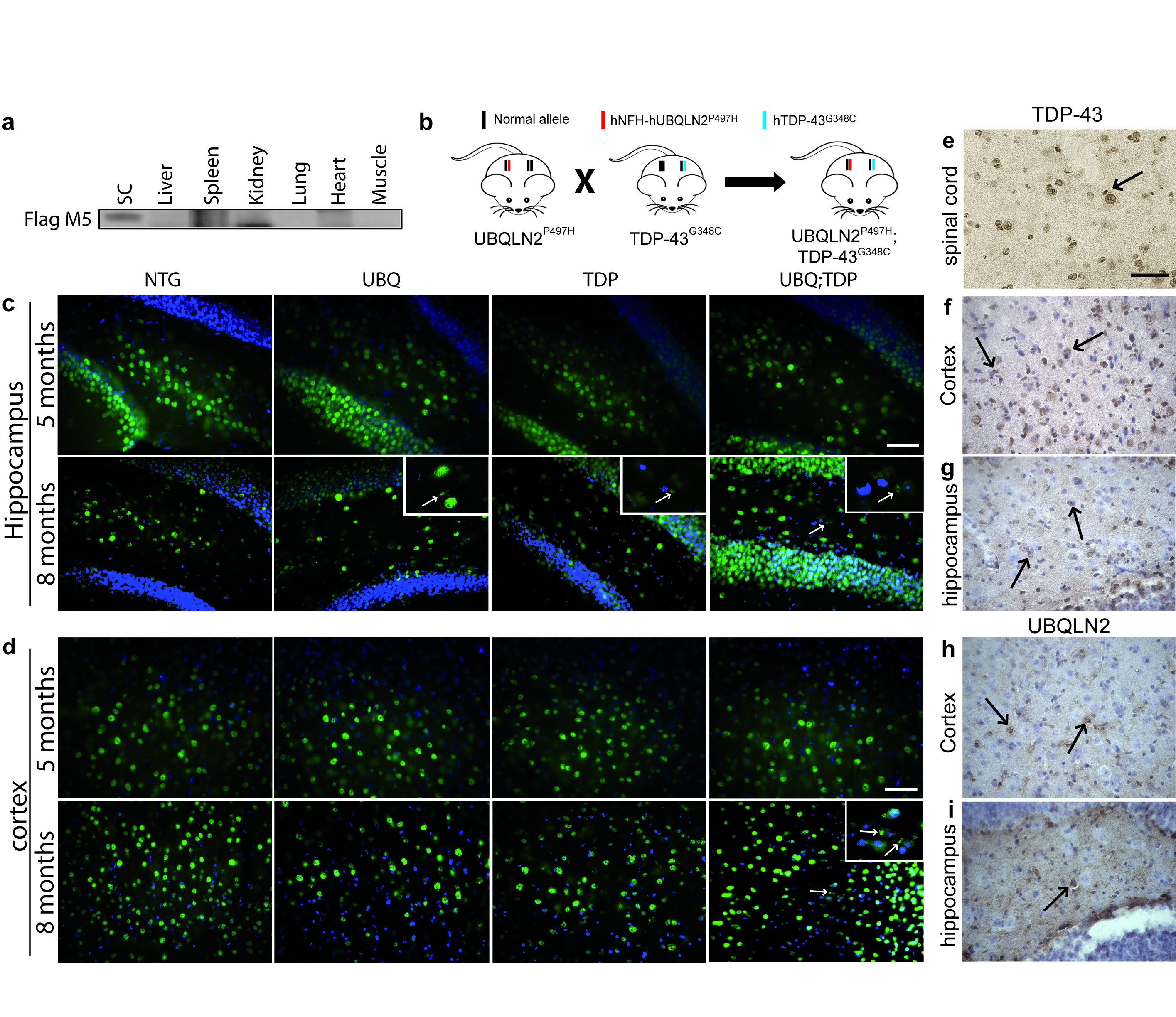

Supplement: Supplementary file 1 — (JPG 5603 kb) [file 12035_2018_1411_MOESM1_ESM.jpg]

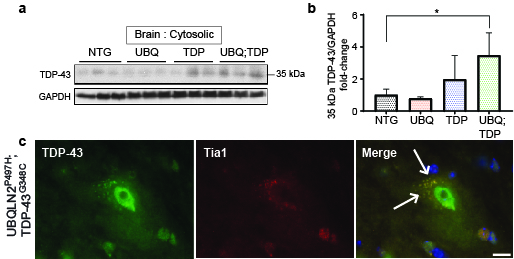

Supplement: Supplementary file 2 — (JPG 193 kb) [file 12035_2018_1411_MOESM2_ESM.jpg]

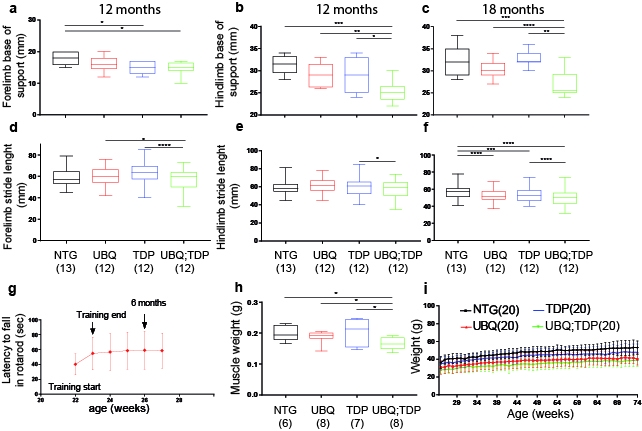

Supplement: Supplementary file 3 — (JPG 249 kb) [file 12035_2018_1411_MOESM3_ESM.jpg]

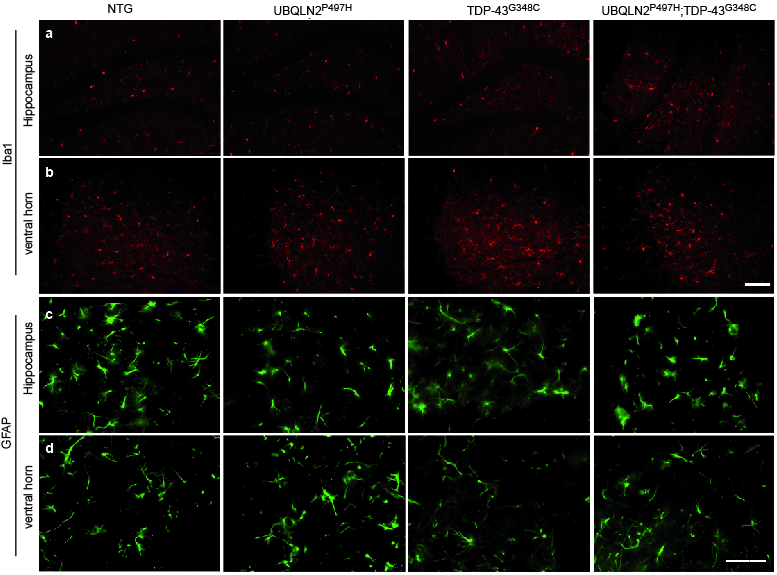

Supplement: Supplementary file 4 — (JPG 966 kb) [file 12035_2018_1411_MOESM4_ESM.jpg]
